# Supplementary material for: Functional Response of Four Phytoseiid Mites to Eggs and First-Instar Larvae of Western Flower Thrips, Frankliniella occidentalis
Source: Insects. 2024 Oct 14;15(10):803. doi: 10.3390/insects15100803 (PMC11508400; doi:10.3390/insects15100803)
Supplement: Supplementary file 1 [file insects-15-00803-s001.zip › Supplementary.Material.Insects.pdf]

Table S1. Impact of temperature on daily prey consumption by four phytoseiid mites at various densities of *F. occidentalis* first-instar larvae

Phytoseiid species	Temperature	*F. occidentalis* larval density					
		5	10	20	30	40	50
A. largoensis	25	5.00 ± 0.00	8.20 ± 0.36	12.70 ± 0.79	12.90 ± 0.67	12.80 ± 0.83	11.60 ± 0.58
	30	5.00 ± 0.00	7.70 ± 0.37	11.40 ± 0.40	12.10 ± 0.57	10.90 ± 0.46	10.10 ± 0.48
U		50	61	66	61.5	75.5	73
P		1	0.387	0.219	0.397	0.055	0.08
P. lenis	25	4.50 ± 0.22	5.50 ± 0.45	5.20 ± 0.49	6.20 ± 0.36	5.50 ± 0.22	4.80 ± 0.44
	30	3.60 ± 0.40	5.80 ± 0.36	6.00 ± 0.42	6.10 ± 0.57	5.80 ± 0.53	5.10 ± 0.35
U		70	44	34	51.5	46.5	41
P		0.111	0.666	0.231	0.938	0.814	0.509
A. swirskii	25	5.00 ± 0.00	8.90 ± 0.28	13.20 ± 0.55	13.10 ± 0.60	12.60 ± 0.48	12.00 ± 0.58
	30	5.00 ± 0.00	8.90 ± 0.35	12.70 ± 0.54	12.10 ± 0.50	10.80 ± 0.66	10.30 ± 0.47
U		50	49	53.5	66	76	71.5
P		1	0.968	0.816	0.233	0.05	0.104
P. cracentis	25	4.80 ± 0.13	8.80 ± 0.42	10.20 ± 0.66	11.70 ± 0.58	11.00 ± 0.42	10.90 ± 0.66
	30	5.00 ± 0.00	8.60 ± 0.37	9.90 ± 0.57	11.10 ± 0.53	10.10 ± 0.67	8.90 ± 0.38
U		40	56	55.5	60.5	65	82
P		0.168	0.666	0.701	0.44	0.264	0.016*

Notes: The values are presented as means ± SE. * P<0.05, ** P<0.01. *** P<0.001. The P-value followed by the number of asterisks indicates the level of statistical significance of the difference (P<0.05, Mann-Whitney U test).

Table S2. Impact of temperature on daily prey consumption
by four phytoseiid mites at various densities of *F. occidentalis* eggs

Phytoseiid species	Temperature	*F. occidentalis* egg density					
		**4-6**	**8-12**	**18-22**	**28-32**	**38-42**	**48-52**
A. largoensis	25	0.20 ± 0.13	1.40 ± 0.22	2.80 ± 0.29	4.70 ± 0.45	4.60 ± 0.48	4.90 ± 0.43
	30	0.20 ± 0.13	1.80 ± 0.13	3.40 ± 0.31	5.70 ± 0.37	6.20 ± 0.29	6.40 ± 0.34
	U	50	34	33	30	18.5	21
	P	1	0.164	0.189	0.128	0.016*	0.027*
P. lenis	25	0.10 ± 0.10	0.60 ± 0.16	1.30 ± 0.30	2.10 ± 0.23	2.40 ± 0.22	2.40 ± 0.27
	30	0.20 ± 0.13	0.80 ± 0.23	2.20 ± 0.25	3.40 ± 0.27	3.70 ± 0.21	3.90 ± 0.28
	U	45	44	25	13	10	10.5
	P	0.583	0.645	0.053	3.76×10^{-3}**	1.52×10^{-3}**	2.07×10^{-3}**
A. swirskii	25	0.20 ± 0.13	0.90 ± 0.23	2.50 ± 0.31	4.20 ± 0.20	4.40 ± 0.27	4.50 ± 0.17
	30	0.20 ± 0.13	1.50 ± 0.22	3.10 ± 0.28	4.90 ± 0.23	5.80 ± 0.33	6.10 ± 0.31
	U	50	28	32.5	20	12	4.5
	P	1	0.081	0.175	1.66×10^{-2}*	2.91×10^{-3}**	3.99×10^{-4}***
P. cracentis	25	0.20 ± 0.13	1.40 ± 0.16	2.70 ± 0.26	4.30 ± 0.37	4.30 ± 0.33	4.50 ± 0.31
	30	0.20 ± 0.13	1.50 ± 0.17	2.90 ± 0.31	4.60 ± 0.31	5.10 ± 0.35	5.20 ± 0.25
	U	50	45	45	44.5	30.5	2.579
	p	1	0.693	0.715	0.692	0.129	0.108

Notes: The values are presented as means ± SE. * P<0.05, ** P<0.01. *** P<0.001. The P-value followed by the number of asterisks indicates the level of statistical significance of the difference (P<0.05, Mann-Whitney U test).

Table S3. Impact of prey type on daily prey consumption of four phytoseiid mites at various densities of *F. occidentalis* eggs or first-instar larvae at 25°C

Phytoseiid species	Prey	*F. occidentalis* density (egg/larval)					
		**4-6/5**	**8-12/10**	**18-22/20**	**28-32/30**	**38-42/40**	**48-52/50**
A. largoensis	Egg	0.20 ± 0.13	1.40 ± 0.22	2.80 ± 0.29	4.70 ± 0.45	4.60 ± 0.48	4.90 ± 0.43
	Larva	5.00 ± 0.00	8.20 ± 0.36	12.70 ± 0.79	12.90 ± 0.67	12.80 ± 0.83	11.60 ± 0.58
U		0	0	0	0	0	0
P		3.29×10^{-5}***	1.41×10^{-4}***	1.56×10^{-4}***	1.68×10^{-4}***	1.72×10^{-4}***	1.69×10^{-4}***
P. lenis	Egg	0.10 ± 0.10	0.60 ± 0.16	1.30 ± 0.30	2.10 ± 0.23	2.40 ± 0.22	2.40 ± 0.27
	Larva	4.50 ± 0.22	5.50 ± 0.45	5.20 ± 0.49	6.20 ± 0.36	5.50 ± 0.22	4.80 ± 0.44
U		0	0	0	0	0	6
P		6.67×10^{-5}***	1.34×10^{-4}***	2.08×10^{-4}***	1.46×10^{-4}***	1.22×10^{-4}***	7.84×10^{-4}***
A. swirskii	Egg	0.20 ± 0.13	0.90 ± 0.23	2.50 ± 0.31	4.20 ± 0.20	4.40 ± 0.27	4.50 ± 0.17
	Larva	5.00 ± 0.00	8.90 ± 0.28	13.20 ± 0.55	13.10 ± 0.60	12.60 ± 0.48	12.00 ± 0.58
U		0	0	0	0	0	0
P		3.29×10^{-5}***	1.43×10^{-4}***	1.50×10^{-4}***	1.45×10^{-4}***	1.41×10^{-4}***	1.17×10^{-4}***
P. cracentis	Egg	0.20 ± 0.13	1.40 ± 0.16	2.70 ± 0.26	4.30 ± 0.37	4.30 ± 0.33	4.50 ± 0.31
	Larva	4.80 ± 0.13	8.80 ± 0.42	10.20 ± 0.66	11.70 ± 0.58	11.00 ± 0.42	10.90 ± 0.66
U		0	0	0	0	0	0
P		6.16×10^{-5}***	1.29×10^{-4}***	1.56×10^{-4}***	1.58×10^{-4}***	1.59×10^{-4}***	1.51×10^{-4}***

Notes: The values are presented as means ± SE. * P<0.05, ** P<0.01. *** P<0.001.The P-value followed by the number of asterisks indicates the level of statistical significance of the difference (P<0.05, Mann-Whitney U test).

Table S4. Impact of prey type on daily prey consumption of four phytoseiid mites at various densities of *F. occidentalis* eggs or first-instar larvae at 30°C

Phytoseiid species	Prey	*F. occidentalis* density (egg/larval)					
		**4-6/5**	**8-12/10**	**18-22/20**	**28-32/30**	**38-42/40**	**48-52/50**
A. largoensis	Egg	0.20 ± 0.13	1.80 ± 0.13	3.40 ± 0.31	5.70 ± 0.37	6.20 ± 0.29	6.40 ± 0.34
	Larva	5.00 ± 0.00	7.70 ± 0.37	11.40 ± 0.40	12.10 ± 0.57	10.90 ± 0.46	10.10 ± 0.48
U		0	0	0	0	0	1
P		3.29×10^{-5}***	1.03×10^{-4}***	1.59×10^{-4}***	1.65×10^{-4}***	1.48×10^{-4}***	2.16×10^{-4}***
P. lenis	Egg	0.20 ± 0.13	0.90 ± 0.23	2.20 ± 0.25	3.40 ± 0.27	3.70 ± 0.21	3.90 ± 0.28
	Larva	3.60 ± 0.40	5.80 ± 0.36	6.00 ± 0.42	6.10 ± 0.57	5.80 ± 0.53	5.10 ± 0.35
U		0	0	0	9.5	11.5	21
P		6.67×10^{-5}***	1.49×10^{-4}***	1.54×10^{-4}***	2.05×10^{-3}**	2.80×10^{-3}**	2.33×10^{-2}*
A. swirskii	Egg	0.20 ± 0.13	1.50 ± 0.22	3.10 ± 0.28	4.90 ± 0.23	5.80 ± 0.33	6.10 ± 0.31
	Larva	5.00 ± 0.00	8.90 ± 0.35	12.70 ± 0.54	12.10 ± 0.50	10.80 ± 0.66	10.30 ± 0.47
U		0	0	0	0	1	1
P		3.29×10^{-5}***	1.33×10^{-4}***	1.58×10^{-4}***	1.49×10^{-4}***	1.86×10^{-4}***	2.09×10^{-4}***
P. cracentis	Egg	0.20 ± 0.13	1.50 ± 0.17	2.90 ± 0.31	4.60 ± 0.31	5.10 ± 0.35	5.20 ± 0.25
	Larva	5.00 ± 0.00	8.60 ± 0.37	9.90 ± 0.57	11.10 ± 0.53	10.10 ± 0.67	8.90 ± 0.38
U		0	0	0	0	0.5	0
P		3.29×10^{-5}***	1.25×10^{-4}***	1.58×10^{-4}***	1.64×10^{-4}***	1.82×10^{-5}***	1.54×10^{-4}***

Notes: The values are presented as means ± SE. * P<0.05, ** P<0.01. *** P<0.001. The P-value followed by the number of asterisks indicates the level of statistical significance of the difference (P<0.05, Mann-Whitney U test).
